# Formate Is Required for Growth of the Thermophilic Acetogenic Bacterium *Thermoanaerobacter kivui* Lacking Hydrogen-Dependent Carbon Dioxide Reductase (HDCR)

**DOI:** 10.3389/fmicb.2020.00059

**Published:** 2020-01-31

**Authors:** Surbhi Jain, Helge M. Dietrich, Volker Müller, Mirko Basen

**Affiliations:** Department of Molecular Microbiology and Bioenergetics, Institute of Molecular Biosciences, Goethe University Frankfurt, Frankfurt am Main, Germany

**Keywords:** hydrogen oxidation, carbon dioxide reduction, hydrogen-dependent carbon dioxide reductase, acetogens, thermophiles, *Thermoanaerobacter kivui*, Wood-Ljungdahl pathway

## Abstract

The hydrogen-dependent carbon dioxide reductase is a soluble enzyme complex that directly utilizes hydrogen (H_2_) for the reduction of carbon dioxide (CO_2_) to formate in the first step of the acetyl-coenzyme A- or Wood-Ljungdahl pathway (WLP). HDCR consists of 2 catalytic subunits, a hydrogenase and a formate dehydrogenase (FDH) and two small subunits carrying iron-sulfur clusters. The enzyme complex has been purified and characterized from two acetogenic bacteria, from the mesophile *Acetobacterium woodii* and, recently, from the thermophile *Thermoanaerobacter kivui*. Physiological studies toward the importance of the HDCR for growth and formate metabolism in acetogens have not been carried out yet, due to the lack of genetic tools. Here, we deleted the genes encoding HDCR in *T. kivui* taking advantage of the recently developed genetic system. As expected, the deletion mutant (strain TKV_MB013) did not grow with formate as single substrate or under autotrophic conditions with H_2_ + CO_2_. Surprisingly, the strain did also not grow on any other substrate (sugars, mannitol or pyruvate), except for when formate was added. Concentrated cell suspensions quickly consumed formate in the presence of glucose only. In conclusion, HDCR provides formate which was essential for growth of the *T. kivui* mutant. Alternatively, extracellularly added formate served as terminal electron acceptor in addition to CO_2_, complementing the growth deficiency. The results show a tight coupling of multi-carbon substrate oxidation to the WLP. The metabolism in the mutant can be viewed as a coupled formate + CO_2_ respiration, which may be an ancient metabolic trait.

## Introduction

Acetogenic bacteria thrive on the production of acetic acid from H_2_ + CO_2_. As such, they are abundant in the environment (Drake et al., 2008), since they link primary fermenters and aceticlastic methanogens in the anoxic food chain (Schink and Stams, 2006). The metabolism of acetogens can be separated into three parts: substrate oxidation (oxidative branch), disposal of reducing equivalents (reductive branch) and redox balancing by electron-bifurcating hydrogenase and/or ferredoxin (Fd)-oxidizing, partly energy-conserving enzyme complexes (Schuchmann and Müller, 2016). H_2_ as electron donor in chemolithotrophic metabolism is primarily oxidized by an electron-bifurcating hydrogenase (Schuchmann and Müller, 2012) providing NADH and reduced ferredoxin (Fd_red_) for CO_2_ reduction. The terminal electron accepting pathway is the presumably ancient Wood-Ljungdahl pathway (WLP; Ljungdahl, 1986; Wood et al., 1986), which is also important in anabolism, since a fraction of its product, acetyl-coenzyme A, is provided for growth. Energy conservation is tightly coupled to redox balancing, since a part of the Fd_red_ is oxidized by energy-conserving membrane-bound enzyme complexes (Schuchmann and Müller, 2014), by the Rnf complex, as present in e.g., *Acetobacterium woodii* (Biegel and Müller, 2010), or by energy-converting hydrogenases Ech encoded in the genome of *Moorella thermoacetica* (Pierce et al., 2008) or as present in *Thermoanaerobacter kivui* (Hess et al., 2014; Schoelmerich and Müller, 2019).

The first step in the methyl-branch of the WLP is the reduction of CO_2_ to formate, which is catalyzed by a formate dehydrogenase (FDH). In some acetogenic microorganisms, FDH occurs in a complex with a hydrogenase and two small subunits to form a hydrogen-dependent carbon dioxide reductase (HDCR) (Schuchmann and Müller, 2013). A distinct property of the soluble enzyme complex is the direct use of H_2_ as electron donor. Therefore, HDCR is the second H_2_-oxidizing hydrogenase in acetogenic catabolism besides the electron-bifurcating hydrogenase. The enzyme complex has been purified from two acetogenic bacteria, the mesophile *Acetobacterium woodii* (Schuchmann and Müller, 2013) and the thermophile *Thermoanaerobacter kivui* (Schwarz et al., 2018). HDCR contained four subunits, a hydrogenase, a formate dehydrogenase and two small subunits bearing iron-sulfur clusters, which are likely to be involved in electron transfer from the hydrogenase to the formate dehydrogenase. HDCR catalyzes formate-dependent hydrogen formation, as determined by the concentrations of H_2_, CO_2_ and formate. The reduction of CO_2_ to formate with hydrogen as electron donor is close to the thermodynamic equilibrium (E_0_′ [CO_2_/formate] = –432 mV; E_0_′ [2 H^+^/H_2_] = –414 mV), and HDCR catalyzed formate oxidation to CO_2_ at comparable rates. Moreover, the reactions were catalyzed with high turnover frequencies (TOF) of up to 101,600 h^–1^ in *A. woodii* and 10,000,000 h^–1^ in *T. kivui*, making HDCRs promising candidate enzymes for biotechnological applications such as H_2_ storage or H_2_ release from stored formate (Pereira, 2013; Schuchmann and Müller, 2013; Müller, 2019).

**FIGURE 1:**
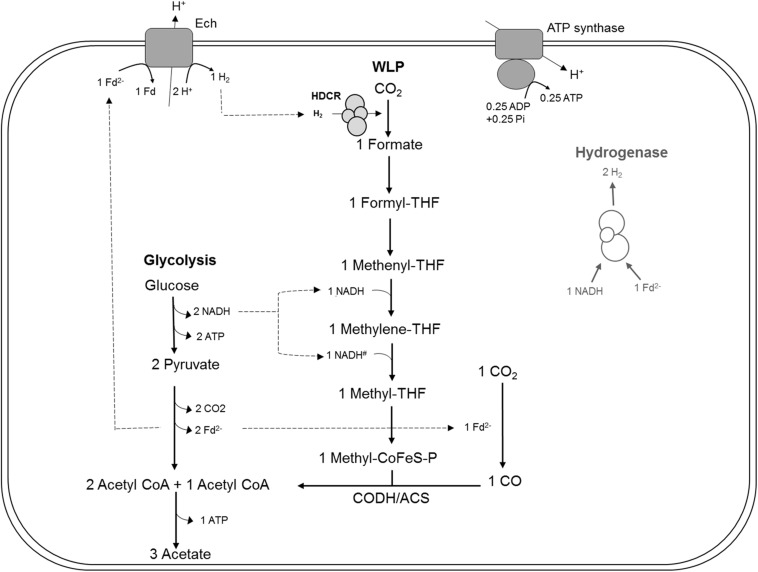
Bioenergetic model of glucose metabolism in *T. kivui*, modified after Hess et al. (2014), highlighting the assumed role of the hydrogen-dependent carbon dioxide reductase (HDCR) in heterotrophic growth. Re-oxidation of ferredoxin (Fd_red_) may be catalyzed by the electron-converting hydrogenase (Ech) complex, and evolved hydrogen (H_2_) may be re-oxidized by HDCR. Alternatively, it may be catalyzed in conjunction with NADH re-oxidation by electron-bifurcating hydrogenase HydABC (transparent, on the right), putatively making HDCR dispensable during heterotrophic growth of *T. kivui*. The stoichiometry of Ech and ATP synthase have been assumed to be 1 proton translocated per one H_2_ evolved in the methanogen *Methanosarcina mazei* (Welte et al., 2010), in the thermophilic archaeon *Pyrococcus furiosus* (Sapra et al., 2003) and in *T. kivui* (Schoelmerich and Müller, 2019). The ATP synthase of *T. kivui* is proton-dependent (Hess et al., 2014), and the ratio of 0.25 ATP per proton translocated is supported by thermodynamic calculations in acetogens (Schuchmann and Müller, 2014). #, The NADH-dependence of methylene-THF reductase has been assumed, based on the model of *A. woodii* and no biochemical evidence for electron-bifurcation. CoFeS-P, Corrinoid iron-sulfur protein.

Many acetogens are metabolically versatile, and able to utilize electron donors other than H_2_, such as sugars, products of primary fermentations such as alcohols or C1 compounds (methanol, formate, CO), or methylated nitrogen compounds such as glycine betaine, in addition to H_2_ (Diekert and Wohlfarth, 1994; Schuchmann and Müller, 2016). Here, we studied the function of the HDCR complex *in vivo* using genetic tools (Basen et al., 2018), with focus on its role in the catabolic conversion of multi-carbon substrates. Our hypotheses were that (i) HDCR is essential in formate oxidation during growth on formate as sole substrate, since it is the only FDH annotated in the genome (Hess et al., 2014), and (ii) in heterotrophic metabolism, HDCR, its product formate and the Wood-Ljungdahl pathway are essential unless electrons are disposed elsewhere, e.g., as H_2_ through the reaction of the electron-bifurcating hydrogenase (Figure 1). Interestingly, the generation of a mutant, strain TKV_MB013, that lacked the genes predicted to encode for the subunits of HDCR, was only possible if formate was supplied in addition to sugars, and the phenotype of the strain was characterized in detail.

## Results

### Generation of a HDCR Genes Deletion Mutant

**FIGURE 2:**
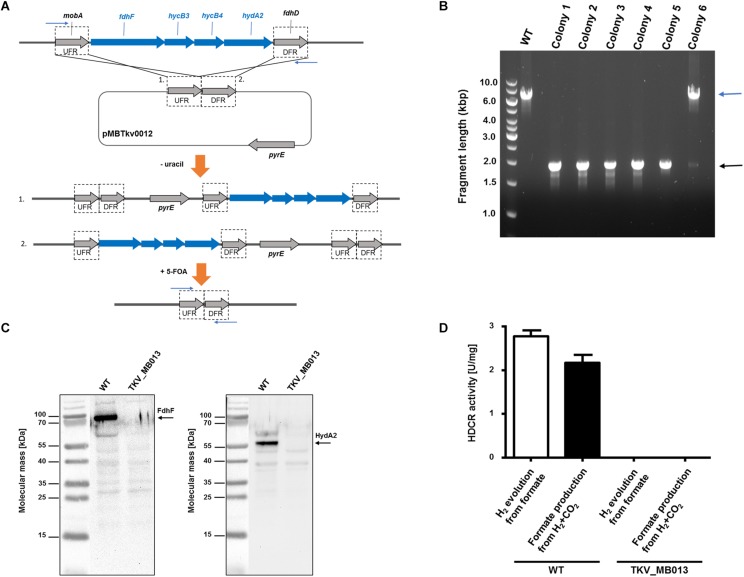
Deletion of the genes encoding HDCR, *fdhF* (TKV_c19990), *hycB3* (TKV_c19980), *hycB4* (TKV_c19970) and *hydA2* (TKV_c19960). **(A)** Strategy for deletion using plasmid pMBTkv0012. 1. and 2. refer to insertion of the plasmid in the first round of selection on uracil prototrophy at the upstream flanking region (UFR) or at the downstream flanking region (DFR), respectively. **(B)** DNA fragments separated by agarose gel electrophoresis after PCR amplification of the HDCR gene locus, using primers outside the flanking regions (indicated as blue arrows). wild type, WT; colonies 1–6). **(C)** Detection of the HDCR subunits FdhF (left side) and HydA2 (right side) in cell free extracts of *T. kivui* DSM2030 (wild type, WT) or of *T. kivui* TKV_MB013. Cells were grown in complex medium (Leigh et al., 1981) with 28 mM glucose and 50 mM formate. 40 μg of cytoplasmic fractions were separated *via* denaturing gel electrophoresis, and then transferred to a nitrocellulose membrane. The presence of FdhF and HydA2 was determined immunologically with antibodies raised against corresponding His-tagged proteins, purified by affinity chromatography. For comparison of the molecular masses, the PageRuler^®^ Prestained Protein Ladder (Thermo Scientific, Dreieich, Germany; left side of both images) was loaded onto the same gel, a picture of the membrane with and without chemiluminescence was taken (ChemoStar, INTAS, Göttingen, Germany), and both images were assembled using the ChemoStar TS software (INTAS, Göttingen, Germany). **(D)** Hydrogen evolution from formate (white bars) and formate formation from H_2_ + CO_2_ (black bars) of cytoplasmic fractions from *T. kivui* wild type (WT) and the HDCR deletion strain (*T. kivui* TKV_MB013). Cells were grown in complex medium with 28 mM glucose and 50 mM formate, and harvested in the late exponential growth phase. 0.3 mg of cytoplasmic protein was incubated in the reaction buffer (100 mM HEPES, 20 mM MgSO_4_, 0.0001% resazurin, 0.5 mM DTE, pH 7.0) at 64°C, and formate (150 mM) or H_2_ + CO_2_ (80:20 [v:v], 1.1 × 10^5^ Pa) were added as substrate. H_2_ production from formate was measured in the gas phase, and formate production from H_2_ + CO_2_ was measured in the liquid phase (*n* = 3).

As HDCR likely fulfills an essential function during growth on formate or on H_2_ + CO_2_, but potentially not during growth on sugars (Figure 1), we aimed to delete the genes encoding the four subunits forming the active enzyme, *fdhF* (TKV_c19990), *hycB3* (TKV_c19980), *hycB4* (TKV_c19970), and *hydA2* (TKV_c19960) in *T. kivui* (Schwarz et al., 2018). These consecutive HDCR genes are part of a gene cluster that also contains a fifth gene, *fdhD* (TKV_c19950), presumably encoding a formate dehydrogenase maturation protein (Schwarz et al., 2018). *FdhD* was not deleted, since it was not identified as part of the enzyme complex in *T. kivui*. For ease of understanding, we refer to the four targeted genes *fdhF, hycB3, hycB4*, and *hydA2* as the HDCR genes in the following. In order to create the HDCR genes deletion in *T. kivui*, plasmid pMBTkv012 was designed (Supplementary Figure S1), carrying approximately 1000 bp regions flanking the HDCR genes. Apart from these upstream (5′) and downstream (3′) flanking regions (UFR and DFR, respectively), the plasmid also contained the *pyrE* cassette as selectable marker, to be introduced into the *pyrE*-deficient uracil-auxotrophic strain TKV_MB002. The genetic system has been described in detail recently (Basen et al., 2018). In brief, we selected for uracil-prototrophs in the first selection round, and for the loss of the plasmid including *pyrE* with 5-fluoroorotic acid (5-FOA) in the second round of selection (Figure 2A), as described previously (Basen et al., 2018). Initially, we used glucose as only substrate, but after screening >50 colonies, we did not obtain any mutant lacking the HDCR gene cluster. In a second approach, we added formate (50 mM) in addition to glucose during the selection as it is the product of HDCR (Figure 1). We then obtained five genotypically “clean” HDCR deletion mutants out of 6 screened colonies/isolates as verified by PCR analysis after the second round of selection (Figure 2B), while the gene locus in the 6th picked colony likely reverted to the wild type gene locus. The markerless deletion of the genes encoding HDCR in mutant 5 was verified by sequencing and immunoblotting (Figure 2C), and the mutant was designated *T. kivui* strain TKV_MB013 (Δ*pyrE*, Δ*fdhF hycB3 hycB4 hydA2*). Unlike cell-free extracts of the wild type, cell-free extracts of strain TKV_MB013 neither carried out H_2_-dependent formate production nor formate-dependent H_2_ production (Figure 2D), an activity specific to the HDCR from *T. kivui* or *A. woodii* (Schuchmann and Müller, 2013; Schwarz et al., 2018). Taken together, all genetic and biochemical evidence suggest that *T. kivui* strain TKV_MB013 was devoid of the genes encoding HDCR.

### Formate Is Essential for Growth of the HDCR Deletion Mutant

**FIGURE 3:**
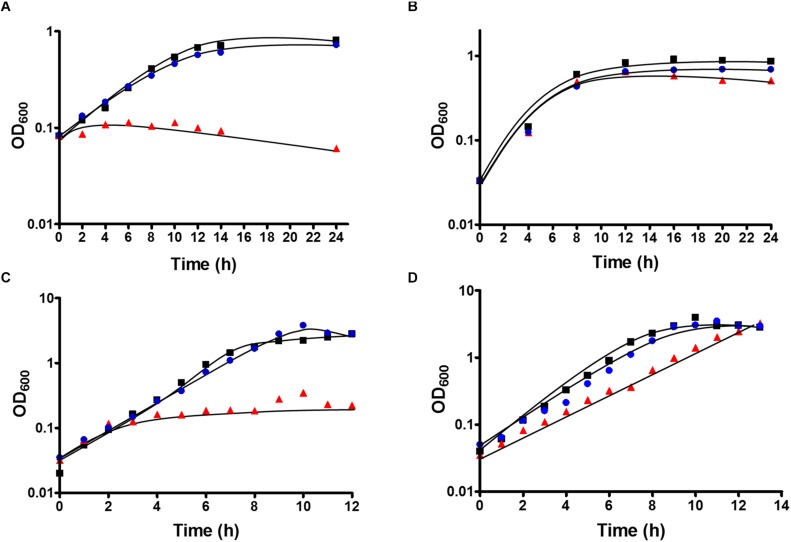
Growth of *T. kivui* strain TKV_MB013 on **(A)** H_2_ + CO_2_ (80:20 [v:v], 2 × 10^5^ Pa), **(B)** H_2_ + CO_2_ (80:20 [v:v], 2 × 10^5^ Pa) + 50 mM formate **(C)** 25 mM glucose or **(D)** 25 mM glucose + 50 mM formate. The cells were grown at 65°C in 100 ml serum bottles containing 25 ml of complex media under an atmosphere of N_2_:CO_2_ (80:20 [v:v], 1.1 × 10^5^ Pa), unless H_2_ + CO_2_ were provided as growth substrates. TKV_MB013, red triangles; TKV_MB013 plus re-introduced HDCR genes in a different genome location, blue circles; or wild type, black squares. The growth experiments were performed in biological triplicates, and a representative growth curve is shown.

After deletion of the HDCR gene cluster and the absence of the protein in strain TKV_MB013 was confirmed, we analyzed the growth phenotype of the strain on all different substrates with and without formate as electron acceptor in addition to CO_2_. Initially, we tested its ability to utilize formate as sole electron donor. As expected, the strain did not grow when 300 mM formate was supplied as sole electron donor (Table 1) due to the absence of HDCR, the sole formate oxidizing enzyme encoded in the genome. The wild type grew to an optical density (OD_600_) of 0.22 ± 0.017. Second, we tested the ability of strain TKV_MB013 to grow chemolithoautotrophically with H_2_ + CO_2_, and this was also not observed (Figure 3A), due to the absence of HDCR as essential formate providing enzyme. In contrast, the wild type grew to an OD_600_ of 0.57 ± 0.02. To indisputably assign this growth deficiency to the loss of the HDCR genes, an ectopic insertion of the wild type genes *fdhF*, *hycB3*, *hycB4*, and *hydA2* into the genome of the mutant strain TKV_MB013 was performed, resulting in strain TKV_MB019. The genes were inserted in between the convergent genes TKV_c24500 and TKV_c24520. The addition of the HDCR genes, controlled by the presumably strong promoter of the S-layer protein of *T. kivui*, complemented the growth effect, therefore we conclude that formate, provided by HDCR activity, is essential for chemolithoautotrophic growth of *T. kivui* on H_2_ + CO_2_. This was expected since formate is an intermediate in the methyl branch of the WLP. To prove this hypothesis, we added formate in addition to H_2_ + CO_2_. In that experiment, formate represented the electron acceptor in the methyl branch of the WLP, and indeed, it supported growth of strain TKV_MB013 on H_2_ + CO_2_ (Figure 3B), with a similar growth rate and final OD_600_ as the wild type.

**TABLE 1:** Average maximal optical densities (OD_600_) from stationary phase cultures of *T. kivui* DSM2030, the mutant *T. kivui* TKV_MB013 lacking the genes encoding HDCR, and its daughter strain *T. kivui* TKV_MB019, with the HDCR encoding genes re-introduced into the genome.

**Substrate used**	**DSM2030**	**TKV_MB013**	**TKV_MB019**
No substrate	n.d.	0.04 ± 0.01	n.d.
25 mM Glucose	2.64 ± 0.11	0.2 ± 0.017	2.4 ± 0.09
25 mM Glucose +	2.86 ± 0.14	3.3 ± 0.1	2.34 ± 0.20
50 mM Formate			
H_2_ + CO_2_ (1 bar)	0.57 ± 0.02	0.02 ± 0.002	0.48 ± 0.004
H_2_ + CO_2_ (1 bar) +	0.8 ± 0.02	0.49 ± 0.01	0.39 ± 0.02
50 mM Formate			
25 mM Mannitol	2.46 ± 0	0.02 ± 0.01	1.08 ± 0.02
25 mM Mannitol +	2.45 ± 0	1.99 ± 0.3	2.4 ± 0.01
50 mM Formate			
300 mM Formate	0.22 ± 0.017	0.01 ± 0	0.16 ± 0.02
50 mM Pyruvate	0.15 ± 0.01	0.03 ± 0	0.15 ± 0.001

*Growth studies were performed in complex media at 65°C until no further increase in OD_600_ was observed. The cells were grown under an atmosphere of N_2_:CO_2_ (80:20 [v:v], 1.1 × 10^5^ Pa), unless H_2_ + CO_2_ were provided as growth substrates (*n* = 2; except for DSM2030 and TKV_MB013 grown on mannitol or on glucose, *n* = 3; n.d., not determined).*

Interestingly, heterotrophic growth on glucose also depended on the presence of the HDCR genes. *T. kivui* strain TKV_MB013 only grew to an OD_600_ of 0.2 (Figure 3C), which was only slightly higher than growth without any substrate added (0.04 ± 0.01). In contrast, the wild type grew to an OD_600_ of 2.64 ± 0.01, which is comparable to what has been observed before (Basen et al., 2018). Again, the ectopic insertion of the HDCR genes into the genome of strain TKV_MB013 complemented the growth deficiency. While the addition of formate did not influence the wild type, formate again stimulated growth of *T. kivui* strain TKV_MB013 (Figure 3D), with a similar OD_600_ of 3.2 reached after 13 h. Interestingly, the addition of formate did not completely restore the growth behavior in strain TKV_MB013, since only a lower growth rate of 0.38 h^–1^ was reached (vs. 0.56 h^–1^ in the wild type). We then tested whether formate addition was essential for growth with all known electron donors for strain TKV_MB013, and this was indeed the case (Table 1). Unlike the wild type, *T. kivui* strain TKV_MB013 neither grew on fructose, mannose (data not shown), pyruvate nor on the recently identified novel substrate mannitol (Moon et al., 2019), to higher optical densities than 0.06, unless formate was added as electron acceptor (Table 1). This unambiguously showed that formate, produced by HDCR, and likely the complete WLP as terminal electron accepting pathway, is essential to growth of the acetogen *T. kivui*.

### Formate Serves as Additional Electron Acceptor in the HDCR Deletion Mutant

**FIGURE 4:**
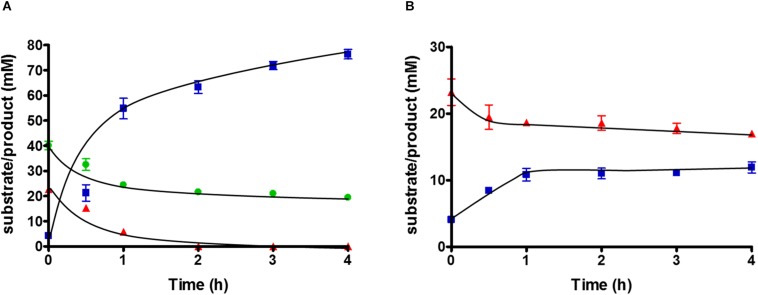
Substrate conversion by 10 ml of 10-fold concentrated cell suspensions of *T. kivui* strain TKV_MB013 (1 mg ml^–1^ protein), pre-grown with glucose and formate, with **(A)** glucose + formate or **(B)** glucose only. Glucose, red triangles; acetate, blue squares; formate, green circles. Experiments were performed in triplicate at 65°C in defined medium, under an atmosphere of N_2_:CO_2_ (80:20 [v:v], 1.1 × 10^5^ Pa).

To study substrate consumption and product formation of *T. kivui* strain TKV_MB013, experiments with concentrated suspensions of resting cells were performed. The cells were concentrated to 10× in defined medium and subjected to a short-termed incubation at 65°C. As expected, when glucose was omitted from the medium and when formate was the sole electron donor, the latter was not consumed, due to the absence of HDCR, and no acetate was produced. When formate was provided as electron acceptor in addition to CO_2_, glucose was completely consumed (22.7 ± 1.9 mM), while the formate concentration decreased from 40.1 ± 2.9 to 19 ± 0.8 mM. Acetate (76.4 ± 3.2 mM) was the sole product (Figure 4A), while only a low concentration of H_2_ was detected in the headspace, corresponding to 0.3 mM, if all H_2_ was dissolved in the medium. Therefore, the results of the cell suspension experiments with the HDCR mutant strain TKV_MB013 reflected the ones from growth experiments. Formate strongly stimulated glucose consumption in the HDCR deficient *T. kivui* strain TKV_MB013, and formate consumption was strictly coupled to glucose consumption. The theoretically assumed stoichiometry based on glucose oxidation to acetate and CO_2_ and concomitant formate and CO_2_ reduction to acetate in the mutant strain, TKV_MB013 is depicted in eq. 1.

(1) 1CH6O12+61.33HCOOH→3.33CHCOOH3+0.66CO+20.66HO2

**FIGURE 5:**
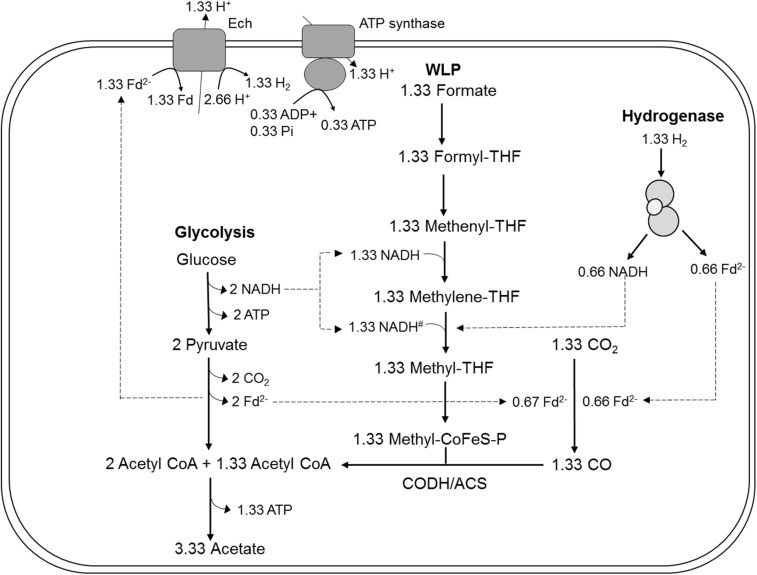
Model for acetogenesis from glucose with formate + CO_2_ as electron acceptors in *T. kivui* TKV_MB013 lacking the genes encoding HDCR. WLP, Wood-Ljungdahl-pathway; HDCR, hydrogen-dependent carbon dioxide reductase; Fd^2–^, reduced ferredoxin; Ech, electron-converting hydrogenase; CoFeS-P, corronoid iron-sulfur protein. For explanations the redox cofactor specificities and of the proposed stoichiometries, please see Figure 1.

Carbon from glucose and formate was stoichiometrically recovered in acetate carbon, under the assumption that one CO_2_ was consumed for each formate consumed (109 ± 14%, *n* = 3). The electron balance, based on oxidation of glucose and reduction of formate and CO_2_ to acetate was nearly closed as well (99 ± 13%, *n* = 3). Therefore, we assume that no other products were present in high concentrations. The measured average acetate to glucose ratio was slightly higher than three (3.2 ± 0.3; *n* = 3), as expected. In conclusion, the observed conversion of glucose and formate to acetate by the HDCR mutant, *T. kivui* strain TKV_MB013 is nearly reflected by theoretically assumed stoichiometry (eq. 1), which reveals that formate served as electron acceptor for the oxidation of H_2_ and organic electron donors (Figure 5).

Since acetogens may dispose electrons from glucose oxidation onto H^+^ to form H_2_, we tested the effect of omitting formate from the cell suspension experiments with *T. kivui* strain TKV_MB013. Glucose was consumed, but only from 23.2 ± 3.4 to 17 ± 0.8 mM, and acetate production stopped at a concentration of 11.9 ± 1.45 mM (Figure 4B). More H_2_ was produced (7.8 ± 0.3 mM, if all hydrogen was dissolved) than in the corresponding experiments with formate. This indicates some reducing equivalents (10.5%) from glucose oxidation were indeed channeled toward H_2_ when formate was omitted. Growth to higher OD_600_ than 0.2 (Figure 3C), however, was not observed.

## Discussion

Acetogens utilize C1-compounds of intermediate redox state such as formate or the methyl groups of methanol, methylamines or methoxylated compounds via the WLP (Kerby et al., 1983; Schuchmann and Müller, 2016). Formate-H_2_ interconversion, catalyzed by formate:H_2_ lyase (FHL), has been studied well in the facultative anaerobe *Escherichia coli* (Mcdowall et al., 2014; Trchounian and Sawers, 2014; Pinske and Sargent, 2016). In contrast to *E. coli*, acetogens utilize formate as sole source of energy and carbon. In principle, acetogens may utilize formate as electron acceptor and electron donor at the same time. Three mols of formate must be oxidized to provide sufficient (6) mols of reductant for the reduction of formate and of CO_2_ to one mol of acetate through the Wood-Ljungdahl pathway (Bertsch and Müller, 2015), according to:

(2) 4HCOOH→1CHCOOH3+1HO2+2CO2

Formate oxidation to CO_2_ is catalyzed by formate dehydrogenase (FDH). The *T. kivui* genome only contains one *fdh* copy, *fdhF* (TKV_c19990). Therefore, the deletion of the HDCR gene cluster including *fdhF* completely abolished its ability to thrive on formate as sole substrate and electron donor (Table 1), which may be different in other acetogens such as *A. woodii* or *Treponema primitia* (Matson et al., 2010) that contain several *fdh* copies, or genes encoding FHLs in addition to *fdh* genes (Poehlein et al., 2015).

Acetogens catalyze formate reduction in the methyl branch of the WLP. Formate is provided by FDH, which is bound to hydrogenase in the HDCR complex in at least two acetogens, *A. woodii* and *T. kivui*. So, in contrast to the membrane-bound FHL that primarily oxidizes formate to CO_2_ in mixed acid fermentation (Pinske and Sargent, 2016), the metabolic function of HDCR is not only formate oxidation but also catabolic CO_2_ reduction to formate in the WLP. Therefore, the ability to use CO_2_ as sole electron acceptor through the WLP was abolished by the HDCR genes deletion. The *T. kivui* mutant lacking the HDCR genes, strain TKV_MB013, depended on the complementation with formate as additional electron acceptor for growth (Figure 3C) and for complete and efficient glucose oxidation (Figure 4A). While our observation has been made - somewhat artificially - with a mutant strain, a similar kind of formate metabolism in native acetogens has been described before, e.g., in *Butyribacterium methylotrophicum*. This bacterium has been reported to utilize CO or H_2_ + CO_2_ and formate simultaneously (Kerby et al., 1983; Kerby and Zeikus, 1987). The acetogen *Acetobacterium woodii* has also been reported to co-utilize formate with CO (Bertsch and Müller, 2015), however, the study had a different focus, since the authors report that CO is only used in the presence of formate. The determined stoichiometries of 1:1.8:1 (formate:CO:acetate) suggest that formate was mainly used as electron acceptor, while CO was used as electron donor (Figure 5). In that scenario, HDCR may be dispensable in the metabolism of *A. woodii*.

The utilization of formate as electron acceptor may indeed be an ancient metabolic trait. Acetogenesis itself supposedly is one of the oldest types of metabolism (Weiss et al., 2016). The first organisms may have thrived on the oxidation of molecular H_2_ with CO_2_, and there are only two groups of organisms that thrive on the conversion of these compounds – methanogenic archaea and acetogenic bacteria. Interestingly, many genes of the WLP essential for CO_2_ fixation in both groups, and the genes essential to acetogenesis, were described as part of the genome of the Last Universal Common Ancestor (Weiss et al., 2016). The role of formate in Early Life, however, is not clear. Strikingly, in acetogens the genes of the WLP are clustered (Poehlein et al., 2015), also in *T. kivui* (Hess et al., 2014), however, e.g., in *A. woodii* (Poehlein et al., 2012) and *Clostridium aceticum* the genes encoding the formate dehydrogenase/HDCR are separate from this cluster. One may speculate that the WLP may have evolved as three independent parts - CO_2_ reduction to formate, formate reduction to a methyl group and the CODH/ACS reaction. In the present study, one of the parts, CO_2_ reduction to formate was removed, which could be complemented by the addition of formate. In an Early Earth environment, organic acids have been reported as prevalent forms of carbon (Amend et al., 2013). Formate has been reported to be thermodynamically more stable than CO_2_ under alkaline conditions, and significant concentrations of formate were found at the alkaline Lost City hydrothermal field (Lang et al., 2010), an environment discussed to have supported the Evolution of Life (Weiss et al., 2016). Thus, formate may have been present as electron acceptor in Early Earth, and a coupled formate + CO_2_ respiration, as described in here, may have allowed the conservation of energy in a primordial environment.

The results also indicate a tight coupling of CO_2_ reduction in the WLP to the oxidation of multi-carbon substrates during heterotrophic growth in *T. kivui*. We initially considered that the HDCR reaction may not be essential for growth on sugars, despite many acetogens have been described as “homoacetogens” such as *Moorella thermoacetica* (Fontaine et al., 1942). The term “homoacetogenesis” refers to the conversion of 1 mol of glucose to 3 mols of acetate (eq. 3), in analogy to homolactate fermentation (Drake et al., 2008). The oxidative part of homoacetate fermentation yields only two mols of acetate, 2 mols of CO_2_ and 8 reducing equivalents (eq. 4), while in the reductive part, catalyzed by the WLP, two mols of CO_2_ are reduced to the third mol of acetate (eq. 5), depleting the reducing equivalents produced in glucose oxidation. This metabolism has been observed during heterotrophic growth of wild type *T. kivui*, with 2.3–3 mols of acetate formed from 1 mol of glucose (Leigh et al., 1981).

(3) 1CH6O12→63CHCOOH3

(4) 1CH6O12+62HO2→2CHCOOH3+2CO+28[H]

(5) 2CO+28[H]→1CHCOOH3+2HO2

In theory, reducing equivalents may take an alternative route, especially, in case that the electron-accepting WLP is impaired. For example, the Fd_red_ and NADH produced in *T. kivui* sugar oxidation may be oxidized by an electron-confurcating hydrogenase (Schut and Adams, 2009). In result, 4 H_2_ were produced per glucose oxidized, in addition to two acetate and two CO_2_ (Figure 1A). That type of metabolism was originally described for the thermophilic bacterium *Thermotoga maritima* (Schut and Adams, 2009), but it is widespread especially among other thermophilic microorganisms. Indeed, in cell suspension experiments with the *T. kivui* HDCR mutant, 7–8 mM of H_2_ (if all headspace H_2_ was dissolved) formed from glucose in the absence of formate (Figure 4B). However, the reducing equivalents present in H_2_ represented only a minor fraction of the total reducing equivalents (10.5%), and the rate of glucose oxidation was much lower than in the experiments with formate present. In growth experiments, cells only reached a low cell density (Figure 3C), even after prolonged incubation times of 1 week (data not shown). Cultures did also not reach higher cell densities when grown with a proportionally larger gaseous headspace (data not shown), indicating that the headspace hydrogen concentration itself was not inhibitory. Therefore, we assume that, while electron-channeling toward H_2_ production was possible in principle, it may not be fast enough to support growth to high cell densities. For example, the electron-bifurcating hydrogenase HydABC of *T. kivui* may be prone to H_2_ oxidation rather than to H_2_ formation, as described for membrane-bound hydrogenases of the hyperthermophilic archaeon *Pyrococcus furiosus* (Mcternan et al., 2014). Alternatively, growth on glucose without a functional WLP or HDCR may be impaired by a non-functional C1-metabolism (since the WLP provides C1-units for anabolic reactions) or by a modified ratio of reduced redox carriers. The latter was observed for *A. woodii*, where a functional Rnf complex was essential for providing Fd_red_ during growth on low-energy heterotrophic substrates such as lactate or ethanol (Westphal et al., 2018).

## Materials and Methods

### Growth Experiments

*Thermoanaerobacter kivui* strain LKT-1 (DSM2030), referred to as wild type, strain TKV_MB002 *(ΔpyrE*, previous name strain TKV002) and strain TKV_MB013 (ΔpyrE ΔTKV_c19960-TKV_c19990) were cultivated under strict anoxic condition at 65°C in complex or defined media as described previously (Weghoff and Müller, 2016; Basen et al., 2018). Complex media contained Na_2_HPO_4_ × 2H_2_O, 50 mM; NaH_2_PO_4_ × 2H_2_O, 50 mM; K_2_HPO_4_, 1.2 mM; KH_2_PO_4_, 1.2 mM; NH_4_Cl, 4.7 mM; (NH_4_)_2_SO_4_, 1.7 mM; NaCl, 7.5 mM; MgSO_4_ × 7 H_2_O, 0.37 mM; CaCl_2_ × 2 H_2_O, 42 μM; Fe(II)SO_4_ × 7 H_2_O, 7.2 μM; KHCO_3_, 54 mM; cysteine-HCl × H_2_O, 3 mM; resazurin, 4.4 μM; 0.2% (w/v) yeast extract, 10 ml/l trace element solution DSM141 and 10 ml/l vitamin solution DSM141. Defined media was prepared similarly, as complex media without addition of yeast extract. The medium was flushed with N_2_:CO_2_ (80:20 [v:v], 1.1 × 10^5^ Pa) before autoclaving. The pH of the medium was 7.5 after flushing. All gases were purchased from Praxair Deutschland GmbH (Düsseldorf, Germany).

Growth experiments were carried out in 20 ml Hungate glass tubes or serum bottles sealed with butyl rubber stoppers under an atmosphere of N_2_:CO_2_ (80:20 [v:v], 1.1 × 10^5^ Pa), unless denoted otherwise (Basen et al., 2018). Usually, a concentration of 25 mM of different organic electron donors such as glucose or mannitol was chosen, and 50 mM formate as electron acceptor. Non-gaseous substrates were added from sterile anoxic solutions. If H_2_ + CO_2_ were used as substrates, tubes were only filled with medium to 1/4 of the volume, and the remaining headspace was replaced with H_2_:CO_2_ (80:20 [v:v], 2 × 10^5^ Pa). To determine the growth behavior, all cultures were inoculated to an optical density of 0.03–0.08 from a pre-culture grown to the exponential growth phase with the same substrate, and then incubated at 65°C under slow shaking. The determination of the cell density was carried out in three biological replicates. Growth in liquid medium was monitored by measuring the optical density at 600 nm. Plating and cultivation on solid media was carried out according to Basen et al. (2018).

### Deletion of HDCR Gene Cluster

Plasmid pMBTkv0012 (Supplementary Figure S1) was used for the deletion of HDCR gene cluster consisting of *fdhF, hycB3, hycB4*, and *hydA2* (TKV_c19960-TKV_c19990). The plasmid was generated by inserting 899 and 1001 bp regions adjacent to the four genes of the cluster (upstream flanking region, UFR, and downstream flanking region, DFR, respectively) into the plasmid pMBTkv005 (Basen et al., 2018). The UFR and DFR were amplified by using the primers NP001 (5′- GCTCG GTACC CGGGG ATCCT AAAGT TTAGT GCATT ACCCC TAAAA TAATG G) and NP002 (5′- CCACT ACCAA CAAAA TTTAA CAAAA CCTCC TCTTA TAACA AAGCA GAAAG G) for UFR, and NP003 (5′- GGAGG TTTTG TTAAA TTTTG TTGGT AGTGG GTTGT AAACA ATCC) and NP004 (5′- GCCGC ATGCC TGCAG GTCGA CTCTA GAGTT ATGTT TAATT TTCTT CCAAC CTCAA CGG) for DFR, followed by the fusion of the PCR products, restriction digest with *Xba*I and *Bam*HI, and by ligation into plasmid pMBTkv005.

*Thermoanaerobacter kivui* Δ*pyrE* was transformed with the plasmid pMBTkv0012, taking advantage of its natural competence for DNA uptake (Basen et al., 2018). The first round of selection was performed in defined media without uracil in the presence of 25 mM glucose + 50mM formate, to select for transformants with the plasmid integrated into the genome. To verify the integration of plasmid pMBTkv012, genomic DNA was extracted and the HDCR gene region was amplified by PCR with the oligonucleotides, NP005 (5′- GATAG GTGAT ACAAT TGAAG TGC) and NP006 (5′- CGCCT CTTGC AAAAC CCG), both binding outside the HDCR gene cluster. Mutants containing the plasmid and growing in the absence of uracil were subjected to a second round of selection as described previously (Basen et al., 2018). Cells were plated on agar with a defined medium containing 50 μM uracil and 5 mM 5-fluoroorotic acid (5-FOA), selecting against the *pyrE* gene. The substrates used were 25 mM glucose + 50mM formate. The genotype of the cells was again checked by using primer pairs NP005/NP006 binding outside and amplifying the complete HDCR gene locus as well as the primer pairs NP001/SJ003 (5′- AGC CGC ATG CCT GCA GGT CGA CTC TAG ATT CAT ATT GAG GCA ATA GTT CAA TAG CC), P9fw (5′- AAA GAT GGT AAA CAG GAA AAG G)/NP007 (5′- CAG GTG TTA AAT CTC CCA AAT), and PBseq10 (5′- GCT CCG GCT ATT AGA GTT TC)/P18brev (5′- GCG TTA TGC CTA CCT ATA TCT TC) each pair leading to the amplification of part of the HDCR gene cluster. The loss of the HDCR gene cluster in the selected *T. kivui* Δ*hdcr* mutant, strain TKV_MB013, was additionally verified by sequencing.

Plasmid pSJ002 (Supplementary Figure S2) was constructed to reintroduce the HDCR gene cluster back into the TKV_MB013 genome, between the convergent genes TKV_c24500 (annoted as AAA family ATPase) and TKV_c24520 (annoted as hydroxylamine reductase), therefore likely not causing polar effects (Basen et al., 2018).

Plasmid pJM006 was used as backbone. Plasmid pJM006 was derived from plasmid pMBTkv007 (Basen et al., 2018), with *pyrE* under control of the promoter controlling gyrase from *Thermoanaerobacter* sp. strain X514, and directly adjacent to the 3′-end, gene Teth514_0627 from *Thermoanaerobacter* sp. strain X514 under control of the promoter of the S-layer protein from *T. kivui*. pJM006 except for *adhE* from *Thermoanaerobacter* sp. strain X514 was amplified by PCR using primers SJ0012 (5′- GAG AAA AAA AGT ATA AAA TTT AAT TTA AAA ATT TCA CAG CAA) and SJ0013 (5′- TTT ACC ATC TTT CAT ACA GTC AAT CCT CCT CCT TG). The HDCR gene cluster of *T. kivui* was amplified by using SJ0010 (5″>- GAG GAG GAT TGA CTG TAT GAA AGA TGG TAA ACA GGA AAA) and SJ0011 (5′- TTT TAA ATT AAA TTT TAT ACT TTT TTT CTC GGT GTA TAT TTA G). The PCR products were then fused to generate the plasmid pSJ002, using Gibson Assembly Mastermix (NEB, Frankfurt/Main, Germany). TKV_MB013 was transformed with plasmid pSJ002. Selection for the transformants was performed by using defined media without uracil in the presence of the substrate 25 mM glucose and 50 mM formate.

### Biochemical Verification of the Absence of HDCR

Immunological detection of the presence or absence of HDCR subunits in cell-free extracts of *T. kivui* strains was performed using antisera containing antibodies specific for the formate dehydrogenase (FdhF, encoded by TKV_c19990) and the hydrogenase subunit HydA2 of HDCR (encoded by TKV_c19960). First, genes encoding both subunits were cloned into plasmids pRT001 (*fdhF*) and pRT002 (*hydA2*). For pRT001, primers PRT1d (5′- TTT GTT TAA CTT TAA GAA GGA GAT ATA CAT ATG AAA GAT GGT AAA CAG G) and PRT2b (5′- CAA GCT TGT CGA CTC AAT GGT GAT GGT GAT GGT GTT TTC CTC CCT TTT CCT TTG C) were used to amplify the *fdhF* fragment, followed by digestion with restriction enzymes *Nde*I and *Sal*I. For pRT002, *hydA*2 fragment was amplified using primers PRT3 (5′- TTT GTT TAA CTT TAA GAA GGA GAT ATA CAT ATG TCT GCA AAT AAA GCT ATA ATT AAT ATA G) and PRT4 (5′- GTG GTG GTG CTC GAG TGC GGC CGC AAG CTT GTC GAC TTA ATG GTG ATG GTG ATG GTG TAC TTT TTT TCT CGG TGT ATA TTT AG), again followed by digestion with *Nde*I and *Sal*I. Fragments were cloned into vector pET21a, which was digested using the same restriction enzymes according to manufacturer’s guidelines (NEB, Frankfurt/Main, Germany). The recombinant, His-tagged versions of FDH or HydA2 were produced in *E. coli* BL21(DE3), purified by affinity chromatography according to standard procedures (Sambrook and Russell, 2001), and sent for rabbit immunization (Davids Biotechnologie, Regensburg, Germany). For Western Blot analysis, 40 μg of *T. kivui* wild type (WT) or TKV_MB013 cell extract was separated *via* denaturing polyacrylamide gel electrophoresis (12%), and immunoblotting onto a nitrocellulose membrane (Protran BA 83; GE Healthcare, United Kingdom) was performed according to standard procedures (Sambrook and Russell, 2001) with goat-anti-rabbit IG, conjugated to horseradish peroxidase (dilution of 1:10,000; Bio-Rad, München, Germany). Rabbit antisera were diluted 1:15,000 (FdhF) and 1:10,000 (HydA2), respectively. The chemiluminescence signal was detected using a chemiluminescence detector (ChemoStar, INTAS, Göttingen, Germany). For comparison of the molecular masses of the detected proteins, two images were recorded of the same membrane, one with and one without chemiluminescence detection; and both images were assembled using the ChemoStar TS software (INTAS, Göttingen, Germany).

Specific HDCR activity in the cell-free extract of *T. kivui* was determined as formate-dependent H_2_ production or as H_2_- dependent formate production from CO_2_ as reported in Schwarz et al. (2018), but at 64°C. Cells for cytoplasmic fraction preparations were harvested in late exponential growth phase and 0.3 mg of cytoplasmic fraction was used for measuring enzymatic activity, respectively. Experiments were conducted in serum bottles and samples for H_2_ or formate measurement were taken about every 2 min. H_2_ evolution from formate was measured in 950 μl reaction buffer (100 mM HEPES, 20 mM MgSO4, 0.0001% Resazurin, 0.5 mM DTE, pH 7.0, N_2_ atmosphere) with 150 mM formate as a substrate. Formate production from H_2_/CO_2_ (80:20 [v:v] 1.1 × 10^5^ Pa) was measured in 5 ml reaction buffer (100 mM HEPES, 20 mM MgSO4, 0.0001% Resazurin, 0.5 mM DTE, pH 7.0) in a two-step enzyme assay. After starting the assay, samples were taken from the liquid phase every 2 min and stored on ice. Determination of formate concentration was then performed using a commercially available formic acid-kit (Boehringer Mannheim/R-Biopharm AG, Mannheim/Darmstadt, Germany). Concentrations of purified proteins or proteins in the cell-free extract were determined as described previously (Bradford, 1976).

### Experiments With Resting Cells

The stoichiometry of metabolite conversion was determined using concentrated suspensions of resting *T. kivui* cells. Initially, 500 ml cultures of *T. kivui* TKV_MB013 (Δ*pyrE*, Δ*fdhF hycB3 hycB4 hydA2*; HDCR deletion mutant) were grown in defined media, in the presence of formate, to the mid exponential phase (OD_600_ of 0.97 to 1.01), and then harvested by centrifugation (Avanti^TM^J-25 and JA-10 Fixed-Angle Rotor; Beckman Coulter, Brea, CA, United States) at 12,700 × *g*, 4°C for 10 min. The supernatant was discarded and cells were re-suspended in 50 ml of defined media. The centrifugation step was repeated, and then, cells were re-suspended again in 50 ml of defined media, and distributed to 10 ml into Hungate tubes. All steps were performed in an anoxic glove box (Coy Laboratory Products, Grass Lake, United States) with an atmosphere of N_2_:CO_2_ (80:20 [v:v], 1.1 × 10^5^ Pa) plus approximately 2% H_2_. The Hungate tubes were closed with butyl rubber stoppers inside the chamber, taken out, and then H_2_ was removed by exchange of the gaseous headspace against N_2_:CO_2_ (80:20 [v:v], 1.1 × 10^5^ Pa). As substrates, 25 mM glucose + 50 mM formate, 25 mM glucose or 50 mM formate were added to the Hungate tubes. The experiment was started by incubation of the concentrated resting cells in a water bath set to 65°C, under slow shaking. 1 ml of subsamples were taken for protein, substrate and product measurements. The protein concentration was determined according to Schmidt et al. (1963).

### Product Analysis

Organic acid and H_2_ production were measured by gas chromatography, in accordance with Weghoff and Müller (2016). Consumption of the substrates glucose and formate was determined by high performance liquid chromatography (HPLC, P680 HPLC Pump, ASI-100 Automated Sample Injector and thermostatted Column Compartment TCC-100, Dionex, Sunnyvale, CA, United States). For the sample preparation, cells were spun down by centrifugation at 13,000 rpm for 5 min and 200 μl of supernatant was filled into 2 ml vials containing 400 μl flat bottom glass insert (Agilent Technologies). A HyperREZ XP Carbohydrate H^+^ ion exchange column (Thermo Fisher Scientific, Waltham, MA, United States) was used for separation. For elution, degassed 5 mM sulfuric acid was used at a flow rate of 0.6 ml/min. The temperature of the oven was set at 65°C. 10 μl of sample was injected by auto-sampler and analyzed with a refractive index detector (RefractoMax 520; Dionex, Sunnyvale, CA, United States) set at 55°C.

## Data Availability Statement

The datasets generated for this study are available on request to the corresponding author.

## Author Contributions

VM and MB designed the study. SJ and HD performed the experiments and prepared the figures. All authors analyzed the data and wrote the manuscript.

## Conflict of Interest

The authors declare that the research was conducted in the absence of any commercial or financial relationships that could be construed as a potential conflict of interest.
