# Supplementary material for: Formate Is Required for Growth of the Thermophilic Acetogenic Bacterium Thermoanaerobacter kivui Lacking Hydrogen-Dependent Carbon Dioxide Reductase (HDCR)
Source: Front Microbiol. 2020 Jan 31;11:59. doi: 10.3389/fmicb.2020.00059 (PMC7005907; doi:10.3389/fmicb.2020.00059)
Supplement: Supplementary file 1 [file Data_Sheet_1.pdf]

Supplementary Material

for the manuscript

Formate Is Required for Growth of the Thermophilic Acetogenic Bacterium *Thermoanaerobacter kivui* Lacking Hydrogen-Dependent Carbon Dioxide Reductase (HDCR)

by

Surbhi Jain[1], Helge M. Dietrich[1], Volker Müller[1] and Mirko Basen[1,2*]

[1]Department of Molecular Microbiology & Bioenergetics, Institute of Molecular Biosciences, Johann Wolfgang Goethe University, Max-von-Laue Str. 9, D-60438 Frankfurt/Main, Germany

[2]Current address: University of Rostock, Institute of Biological Sciences, Albert-Einstein Str. 3, 18059 Rostock, Germany

*** Correspondence:**

Mirko Basen
mirko.basen@uni-rostock.de

1 Supplementary Figures and Tables

1.1 Supplementary Figures

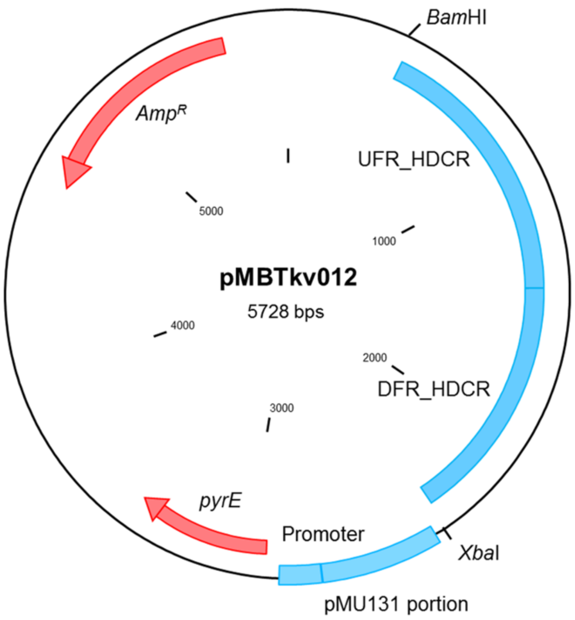

Supplementary Figure 1. Plasmid pMBTkv012. UFR, upstream flanking region; DFR, downstream flanking region; HDCR, genome region encoding the hydrogen-dependent carbon dioxide reductase.

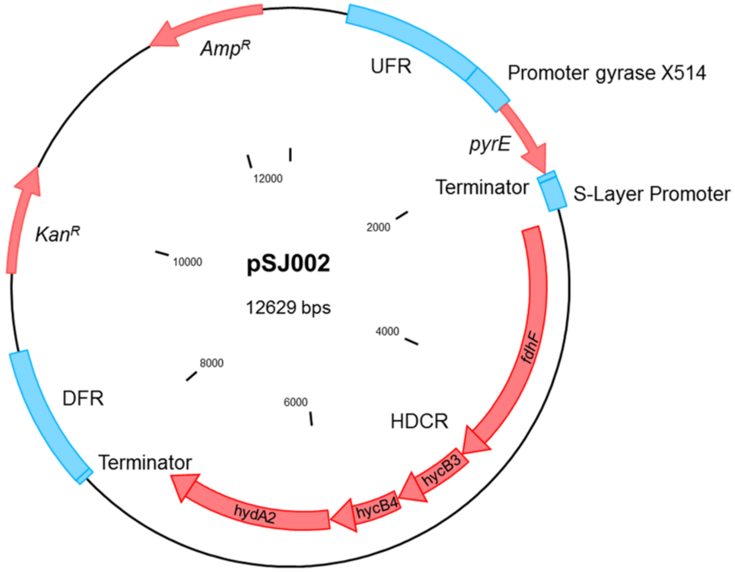

Supplementary Figure 2. Plasmid pSJ002. UFR, upstream flanking region; DFR, downstream flanking region; HDCR, genome region encoding the hydrogen-dependent carbon dioxide reductase; Promoter gyrase X514, putative promoter region of the gene encoding gyrase in *Thermoanaerobacter* sp. strain X514; S-layer promoter, putative promoter of the gene encoding the S-layer protein in *Thermoanaerobacter kivui.*
